# Calcium Imaging of Cortical Neurons using Fura-2 AM

**DOI:** 10.3791/1067

**Published:** 2009-01-19

**Authors:** Odmara L Barreto-Chang, Ricardo E Dolmetsch

**Affiliations:** Department of Neurobiology, Stanford University School of Medicine

## Abstract

Calcium imaging is a common technique that is useful for measuring calcium signals in cultured cells. Calcium imaging techniques take advantage of calcium indicator dyes, which are BAPTA-based organic molecules that change their spectral properties in response to the binding of Ca2+ ions. Calcium indicator dyes fall into two categories, ratio-metric dyes like Fura-2 and Indo-1 and single-wavelength dyes like Fluo-4. Ratio-metric dyes change either their excitation or their emission spectra in response to calcium, allowing the concentration of intracellular calcium to be determined from the ratio of fluorescence emission or excitation at distinct wavelengths. The main advantage of using ratio-metric dyes over single wavelength probes is that the ratio signal is independent of the dye concentration, illumination intensity, and optical path length allowing the concentration of intracellular calcium to be determined independently of these artifacts. One of the most common calcium indicators is Fura-2, which has an emission peak at 505 nM and changes its excitation peak from 340 nm to 380 nm in response to calcium binding. Here we describe the use of Fura-2 to measure intracellular calcium elevations in neurons and other excitable cells.

**Figure:**
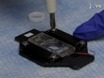


## Protocol

### Cell Culture

Cells can be grown using established techniques but must be plated on #1 glass coverslips coated with a cellular adhesive (like polylysine, polyornithine or laminin) to prevent the cells from detaching or moving during imaging experiments.

### Solutions

Calcium imaging experiments can be performed using a variety of physiological solutions including cell culture media. It is important, however, to make sure that the solutions are free of phenol red, which greatly increases the fluorescent background. We use Tyrodes solution, which is easily made and mimics cerebrospinal fluid, and we supplement it with 0.1% Bovine Serum Albumin. We use depolarization with 60-90 mM potassium chloride to activate voltage gated calcium channels and 1μM Thapsigargin (1 mM stock in DMSO) or 2μM Ionomycin (1 mM stock in DMSO) to activate store operated CRAC channels. It is often convenient to remove calcium from the extracellular solution to show that calcium elevations are due to calcium influx. When removing calcium it is necessary to maintain the total concentration of divalent cations (Mg^2^+ and Ca^2^+) constant. When substituting potassium for sodium it is necessary to maintain the osmotic balance.

Tyrodes solutions:

**Table:** 

	Low Potassium 2mM Ca^2^+Tyrodes (mM)	Low Potassium 0 Ca^2^+ Tyrodes (mM)	High Potassium 2mM Ca^2^+Tyrodes (mM)	High Potassium 0 Ca^2^+Tyrodes (mM)
NaCl	129	129	5	5
KCl	5	5	129	129
CaCl2	2	0	2	0
MgCl2	1	3	1	3
Glucose	30	30	30	30
Hepes	25	25	25	25

Adjust pH to 7.4 with NaOH

#### Loading of Fura-2 calcium dye

We load cells with acetoxy-methyl-ester Fura-2 (Fura-2 AM), which diffuses across the cell membrane and is de-esterified by cellular esterases to yield Fura-2 free acid. The exact parameters for Fura-2 loading vary widely across cell types. We recommend testing various conditions by preparing several loading solutions containing a multiple concentrations of Fura-2 raging from 1- 4 µM, incubating cells in the loading solution for a variety of times from 15 minutes to 2 hours and testing the loading at room temperature and at 37 deg. A simplified protocol for cortical neurons is given below:

1. First, prepare the 1 mM Fura-2 AM stock by adding 50µl of DMSO to a 50µg vial available from Invitrogen. It is important to use dry DMSO packed under nitrogen and it is necessary to remove the DMSO with a needle by puncturing the septum to prevent hydration of the DMSO. After preparing the Fura-2 AM solution keep it in a dark dry place. Fura-2 AM in DMSO is stable at RT for 24 hours and is stable at - 20 degrees in a dry container for several months.
2. Aliquot 2 mls of culture media into a 15 ml conical tube, warm to 37 deg. and add 2µl of Fura-2 AM stock to generate a 1µM Fura-2 AM solution. Vortex the solution vigorously for 1 minute.
3. Transfer the loading solution to a 35 mm tissue culture dish and transfer the coverslip with the cells into the dish.
4. Incubate the neurons at 37 degrees for 30 minutes in a dark incubator. Time the incubation precisely.
5. Prepare a 35 mm dish containing 2 mls of tissue culture media without Fura-2 AM. Remove the coverslip from the loading solution and place in the new dish.
6. Mount the coverslip on the imaging chamber. Remove the coverslip from the 35 mm dish and rapidly mount onto the chamber making sure to prevent drying of the cells. We use an imaging chamber manufactured by Warner Instruments that allows a 10mm coverslip containing the cells to be mounted on the bottom and a second coverslip to be mounted on the top forming a sandwich. The two coverslips are secured with vacuum grease to the chamber and two tubes at either end of the chamber allow for perfusion of solutions through the chamber. The input line is connected to a syringe and the output line is connected to a well that is emptied by a suction line connected to a vacuum trap.

#### The microscope

We use an inverted Nikon Eclipse TE2000-U microscope equipped with a xenon arc lamp (Sutter Instruments), an automated stage (Ludl), an excitation filter wheel (Ludl), and a cooled charge-couple device (CCD) camera (Hamamatsu Orka II). The microscope is controlled by a Macintosh computer running Open Lab software (Improvision). Several other software packages are available for ratiometric imaging. For imaging we use 40, 60 or 100x Nikon Fluor oil immersion objectives with an NA exceeding 1.2.

#### Imaging Protocol

1. Calibrate the microscope stage.
2. Load Tyrodes solution into the input line taking care to prevent the formation of air bubbles.
3. Connect the chamber to the perfusion lines and perfuse Tyrodes solution through the chamber once again, taking care to prevent the formation of bubbles in the chamber
4. Place a drop of oil on the objective, place the chamber on the microscope stage and focus on the cells using transmitted light.
5. Examine the fluorescence of the cells using illumination at 340 and at 380 nm using the eyepieces. Resting cells should be dim at 340 and bright at 380. In general, cells should not be illuminated with UV light for more than 10 or 15 seconds and the intensity of the excitation light should be reduced with a neutral density filter to prevent phototoxicity.
6. Examine the cells using the camera and set the gain and exposure of the camera to generate an image that is close to saturation (but not saturated) when illuminated at 380 nm and well below saturation when illuminated at 340 nm. Keep the exposure below 200ms if possible. Once set, do not change the camera gain or exposure unless you plan to also alter the background, RMin and RMax (see below).
7. Collect an image at each wavelength and use the region of interest (ROI) tool to measure the intensity of the background in a variety of locations in the images for each wavelength.
8. Average the background values and enter the background values into the appropriate locations in the imaging program. The background value will be subtracted from each pixel in the field.
9. Collect a practice ratio image and adjust the Threshold values for each wavelength to generate a ratio image that includes only the cells and not the background and that is not noisy in the region close to the edges of the cells.
10. Set the minimum ratio value (RMin) to be about 10% below the lowest ratio of any cell in the field.
11. Set the RMax to be about 12 times the RMin. Do not change the RMin or RMax between experiments that you plan to compare as it will make the comparison difficult. We seldom change the RMin and Rmax values on our imaging rig.
12. Use the automated stage to find the fields that you plan to image, and record the location of each field using the software. We generally collect five fields of view during an experiment. The automated stage moves to a field, collects a ratio image and moves on to the next field.
13. Set up the time-lapse interval to collect images between 0.1 and 10 seconds apart depending on the type of signals that you expect to see.
14. Start the experiment.
15. When testing the calcium imaging system it is often useful to use stimuli like high potassium tyrodes (65 mM KCl) or ionomycin (2µM) that will cause an intracellular calcium rise and calcium free extracellular solution that will (containing the calcium buffers like EGTA and BAPTA) that will reduce the calcium concentration.

### Analysis

Once the experiment is complete you will want to convert the set of ratio images into time-lapse calcium measurements for individual cells or regions of interest within cells. To do this:

1. Use the region of interest tool (ROI) to define the areas of the image in which you want to measure calcium. It is generally useful to have at least one ROI that covers the cell body of the cell. When defining the ROIs, it is useful to play the movie of the images to verify that the cells do not move during the course of the experiment.
2. Use the software to collect time-lapse ratio measurements for each ROI in each image.
3. Import the ratio measurements into an analysis program. You will need to view the calcium traces for specific cells or ROIs, average multiple cells or ROIs and convert the ratio measurements to intracellular calcium values. We use a set of macros written in Igor Pro which allow us to do all these common analysis tasks but other programs like Excel, though less convenient can also be used.
4. The equation [Ca] = (R-R_min_)/(R_max_-R)Sf*Kd can be used to convert the Fura-2 ratio values to intracellular calcium concentrations. [Ca] is the calcium concentration, R is the Fura-2 340/380 ratio, RMin and RMax are the 340/380 ratios in the absence of calcium or in the presence of a saturating concentration of calcium respectively; and Sf*Kd is the product of the Kd of Fura-2 (approximately 120 nM at RT) and a scaling value. To measure R_Min_, R_Max_ and Sf*Kd it is necessary to perform either an in vivo or an in vitro calibration. In vivo calibration requires a combination of patch clamping and calcium imaging, which can be complicated but is also very precise. In vitro calibration can be done using a home-made calibration chamber that consists of two coverslips separated by a thin coverslip spacer to generate a thin layer of solution in which to perform the calibration. The most convenient way of measuring Fura-2 ratio values as a function of calcium concentrations is to use a calibration kit that contain multiple buffered calcium solutions and Fura-2 free acid. These can be obtained from Invitrogen (Molecular Probes) and contain precise instructions for use.

## Discussion

In this presentation we've gone through all the steps for performing calcium imaging using Fura-2 AM. We used cortical neurons as a model but calcium imaging can be used to measure intracellular calcium in real time in a variety of cells. When doing this procedure it is important to use glass coverslips instead of plastic, since plastic is often fluorescent at ultraviolet wavelengths, which makes imaging difficult. Also, it is important to pre-coat the coverslips with polyornithine and laminin in order to prevent cells from detaching. Finally, it's important to remember to avoid creating air bubbles when perfusing solutions through the imaging chamber.
